# Case report: COVID-19 infection in a pregnant 33-year-old kidney transplant recipient

**DOI:** 10.3389/fmed.2022.948025

**Published:** 2022-08-30

**Authors:** Dorina Supák, Balázs Mészáros, Márta Nagy, Dániel Gáspár, László J. Wagner, Zoltán Kukor, Sándor Valent

**Affiliations:** ^1^Department of Obstetrics and Gynecology, Semmelweis University, Budapest, Hungary; ^2^Department of Molecular Biology, Institute of Biochemistry and Molecular Biology, Semmelweis University, Budapest, Hungary; ^3^Department of Surgery, Transplantation and Gastroenterology, Semmelweis University, Budapest, Hungary

**Keywords:** COVID-19, SARS-CoV-2, kidney transplant, pregnancy, preeclampsia, interleukin-6

## Abstract

Patients facing severe acute respiratory syndrome-coronavirus 2 (SARS-CoV-2) infections with comorbidities, especially patients whose immune system is weakened have higher chances to face severe outcomes. One of the main reasons behind the suppression of the immune system is iatrogenic, in patients who have autoimmune diseases and/or had an organ transplant. Although there are studies that are examining immunocompromised and/or transplanted patients with COVID-19 infection, furthermore there is a limited number of studies available which are dealing with COVID-19 in pregnant women; however, it is unique and is worth reporting when these factors are coexisting. In this study, we present the case of a 33-year-old Caucasian pregnant woman, who had a kidney transplant in 2009 and contracted the SARS-CoV-2 virus on the 26th gestational week, in 2021. After her infection, superimposed preeclampsia was diagnosed and due to the worsening flowmetric parameters, she gave birth to a premature male newborn with cesarean section. Our kidney transplant patient’s case highlights how COVID-19 disease can lead to preeclampsia and artificial termination of gestation.

## Introduction

The incidence of neonatal morbidity, mortality, and preeclampsia are reportedly higher among kidney transplanted pregnant patients and these pregnancies should be deemed high risk and require multidisciplinary monitoring (1, 2).

We present the case of a 26 weeks pregnant, 33-year-old kidney transplanted woman with COVID-19 disease, with whom the following complications occurred during her pregnancy: preeclampsia, intrauterine growth restriction (IUGR), severe fetal vascular malperfusion.

## Case description

A 33-year-old Caucasian, 26 weeks pregnant primipara with a body mass index (BMI) of 24.2 (before the pregnancy 19.3) presented a positive COVID-19 PCR in 2021, at the hospital admission, her symptoms were mild. The patient’s medical history involved acute pyelonephritis (2008), followed by acute necrotizing pancreatitis (2008), leading to sepsis with acute kidney injury. The prolonged sepsis with severe complications led to chronic renal insufficiency, therefore she had a cadaveric kidney transplant surgery in 2009.

In 2011 she was diagnosed with Crohn’s disease.

Her medications before the severe acute respiratory syndrome-coronavirus 2 (SARS-CoV-2) infection were the following: tacrolimus (4.5 mg/day), prednisolone (7.5 mg/day), and she also took a pregnancy food supplement daily, which contained folic acid, metafolin, docosahexaenoic acid (DHA), iodine, B1-, B2-, B6-, B12-, C-, D3-, E-vitamins, biotin, niacin, and pantothenic acid.

After her COVID-19 infection, her medications were complemented with 100 mg/day of aspirin, a.6 ml subcutaneous enoxaparin-sodium injection.

**FIGURE 1:**
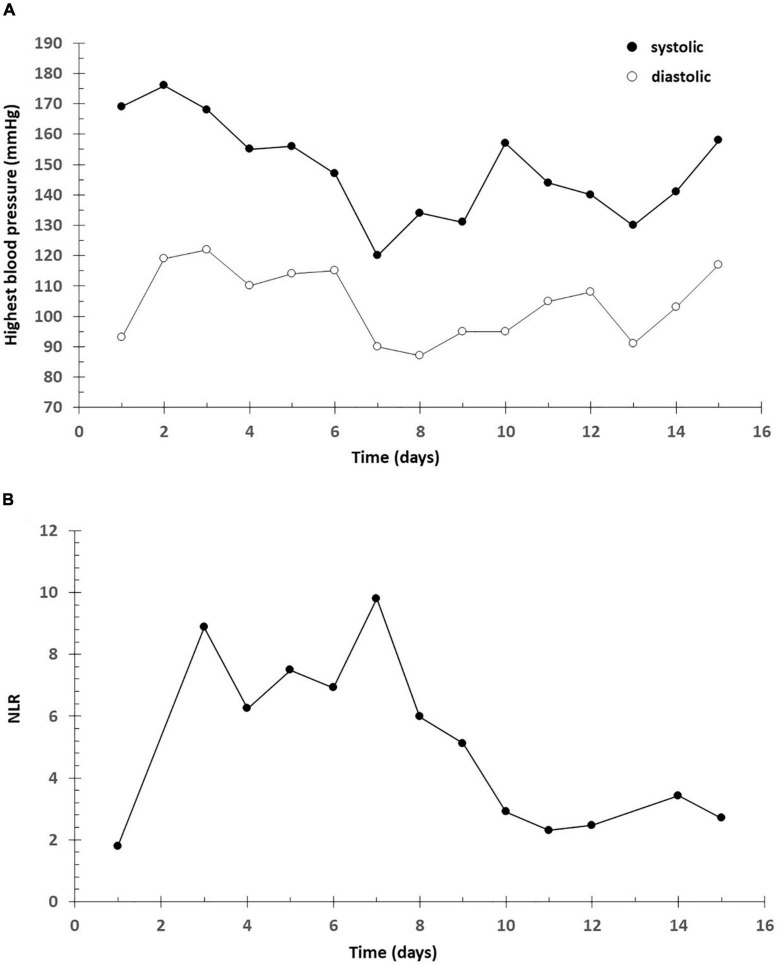
Highest blood pressure. NLR. **(A)** Higher blood pressure (mmHg). Systolic (•) and diastolic (o) blood pressure. **(B)** Neutrophil-Lymphocyte Ratio (NLR).

**FIGURE 2:**
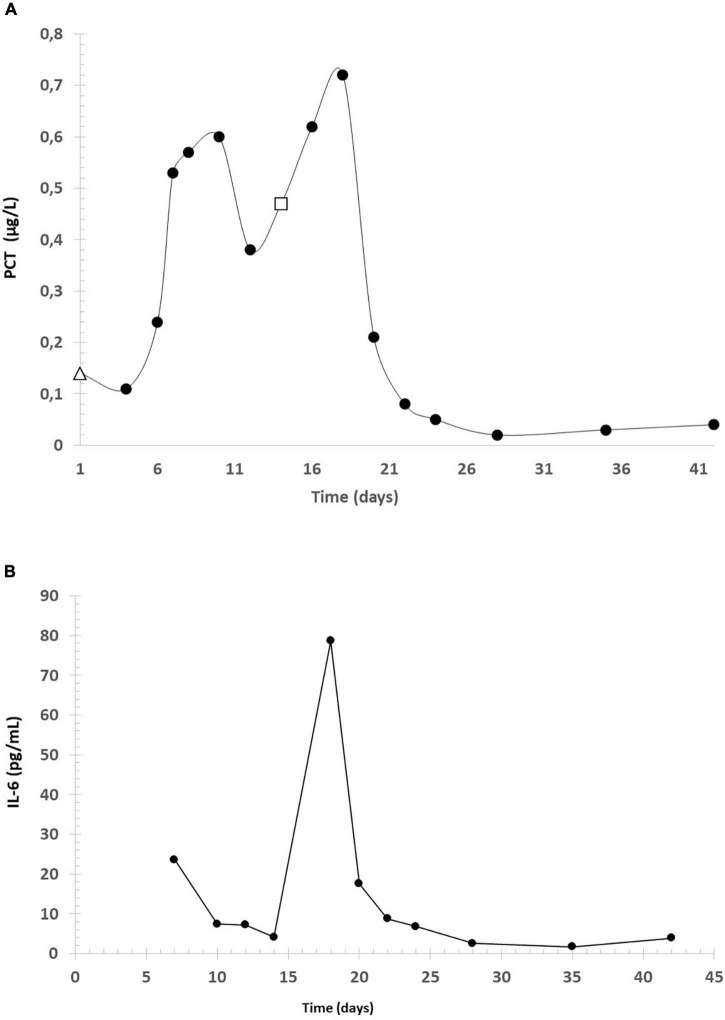
Concentration of PCT and IL-6. **(A)** PCT (which was measured for the first time on the day of the hospital admission–marked with Δ) during the infection, in μg/L. The last data before the birth was marked with □. **(B)** interleukin-6 (IL-6) concentration.

Then, 4 days after her positive COVID-19 test, she was admitted to the hospital with progressively elevating hypertension for the reason that the patient was not able to manage steroid prophylaxis for fetal lung maturation. We marked the day of the hospital admission as day number 1. On this day, laboratory examinations were performed, and they indicated quantitative proteinuria; for the purpose of lung maturation, she received 6 mg of dexamethasone four times in intramuscular injection. The values of laboratory findings are presented in Figures 1, 2 and Supplementary files.

**FIGURE 3:**
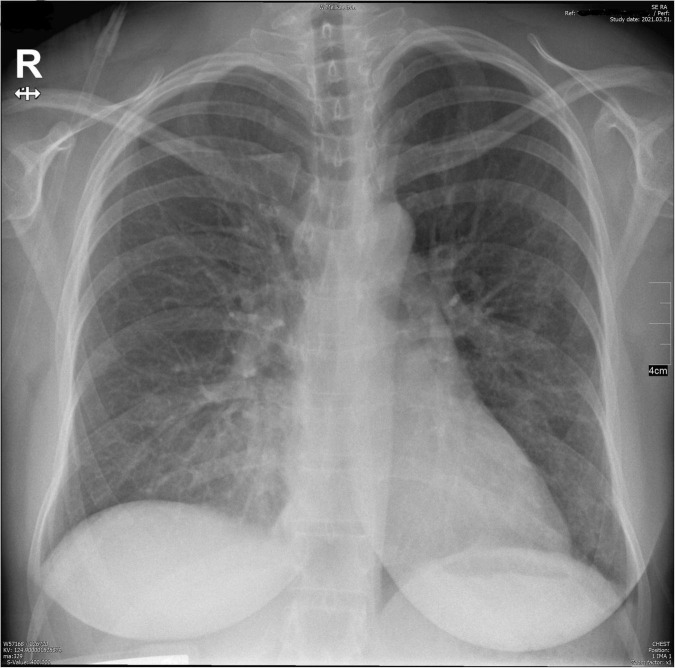
Pulmonary X-ray on the 6th day.

On the 6th day, the pulmonary X-ray did not show large infiltration, but it could not close out mild bronchopneumonia on the right lung (Figure 3). Due to her decreased oxygen saturation, non-invasive ventilation (NIV) was induced with 2–3 L/min flow (between the 5th day and the 10th day). She once received anti-SARS-Cov-2 convalescent plasma therapy as the pilot study of Rodionov et al. (3) also suggests that immunocompromised patients can benefit from plasma high in anti-SARS-CoV-2 IgG. As a result of the mentioned therapies, her respiratory symptoms weakened but her laboratory results remained to confirm superimposed preeclampsia. The platelet count was below the lower limit (150,000 platelets/μL) on the first 9 days of hospitalization. The lowest value was 104,000 platelets/μL (Supplementary files). Thus, hemolysis, elevated liver enzyme levels, and low platelet levels (HELLP) syndrome did not develop.

**FIGURE 4:**
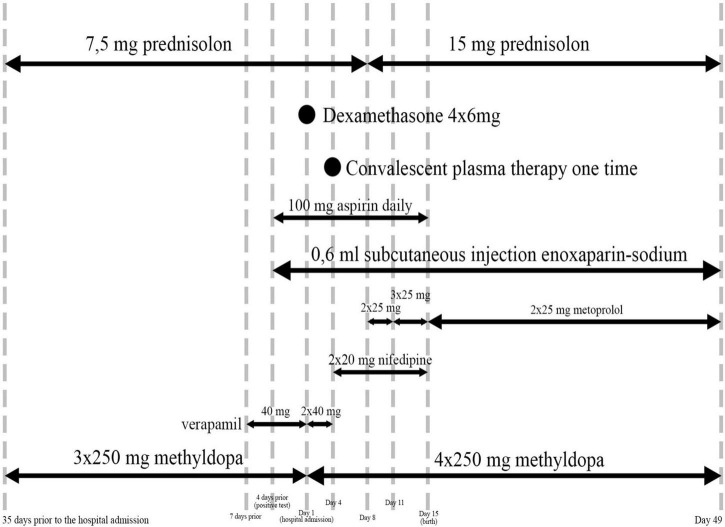
Medications.

Furthermore, 35 days prior to her hospital admission, she was diagnosed with gestational hypertension. Urine had no protein content. Preeclampsia has not yet developed. Hypertension was treated with methyldopa 3 × 250 mg a day, even though it was advised to be taken four times. Then, 40 mg of verapamil was added to her therapy 7 days prior to her hospital admission. In the hospital, she received 2 × 40 mg of verapamil, 4 × 250 mg of methyldopa, and 25 mg of metoprolol. On the 4th day, instead of verapamil, she received 2 × 20 mg of nifedipine, on the 8th day, she received 2 × 25°mg of metoprolol, on the 5th day, she received 3 × 25 mg of metoprolol, and then she continued to take 4 × 250 mg of methyldopa. After giving birth and leaving the COVID-ward, she remained to take 4 × 250 mg of methyldopa and 2 × 25 mg of metoprolol (see Figure 4). Her blood pressure remained high during her pregnancy (with the highest being recorded at 176/119 mmHg), which highlighted the diagnosis of preeclampsia.

Fever was measured on the 5th and 6th day, wherein the highest body temperature was 38.4°C, measured on the morning of the 6th day. Due to the deterioration, a cesarean section was performed on day 15th.

During her observation, an obstetric ultrasound examination was performed daily, which showed IUGR and increasing vascular resistance. The first detection of circulation centralization was on the 12th day, which was 3 days prior to the birth.

After consulting with neonatologists, on the 15th day, which is in the 29th gestational week, a cesarean section was performed because of the following reasons: preeclampsia, IUGR, and increased vascular resistance. On the day of the birth, the umbilical artery showed the signs of the total diastolic stop. The mother gave birth to a premature, living boy who was weighing 990 g, with an Apgar score of 6/8, and a 7.18 pH of umbilical arterial blood.

The neonate’s birth weight was in the 2.5th percentile (990 g), which confirms the ultrasound diagnosis of IUGR.

The mother presented her first negative SARS-CoV-2 PCR test on the day of the birth, and the second one was 2 days later. On the 17th day, the neonate also presented negative tests same two days. After the two negative tests, the mother was replaced from COVID-care-unit to a regular postnatal ward.

After the birth, the neonate was admitted to NICU as he required nCPAP respiration support, and the therapy was needed for 16 days. Even though the highest FiO2 was 30%, and after the 5th day he mostly received air with 21% FiO2, ophthalmology consultants found the progression of retinopathy (ROP 3 (+) zona 2 stages on the right eye, ROP 2 (+) stage on the left eye), and laser therapy was advised.

Cranial ultrasound examination found 2nd stage intraventricular hemorrhage, which was not progrediating.

The child was growing well; after 9 days, his breast milk feeding was built up. He was discharged from the hospital with a weight of 1,830 g.

The mother did not need hospital care after the 49th day but she stayed in the hospital while the newborn was in the NICU. The two were discharged from the hospital on the 63rd day.

## Discussion

The prevalence of preeclampsia and preterm delivery tends to be higher among kidney transplanted patients (4) and according to the latest studies COVID-19 disease can also play an important role in the development of preeclampsia and preeclampsia-like symptoms (5). Since the two conditions were co-existing in the presented case, we think that it could have a synergistic effect in the forming of the clinical characteristics of superimposed preeclampsia, which was one of the reasons behind cesarean delivery in the 29th week.

The INTERCOVID study (which involved 43 institutions in 18 countries) shows that SARS-CoV-2 infected women have a higher risk of delivering their babies preterm, both spontaneously and in an artificially indicated way (6).

This study also indicates that cesarean delivery increases the risk of neonatal COVID-19 infection. Even though there have been reports of vertical transmission, the occurrence was low (7)–such as in this case the newborn presented a negative PCR test and vertical transmission was excluded.

COVID-19 is also a disease that is associated with endothelial dysfunction and injury of the (micro) vascular system which can lead to impaired perfusion of the organs and the fetus (8, 9). This can be a logical explanation for the worsening flowmetric parameters in the presented case.

Even though COVID-19 is characterized as a respiratory disease since its pathomechanism is highly dependent on ACE2 receptors which are expressed in other organ systems, there are studies that highlight that SARS-CoV-2 also can cause damage to the liver (10)–in the case of our patient we detected highly elevated liver enzymes (ASAT and ALAT) during the infection.

In their review, Guney et al. analyzed the relationship between inflammatory markers [for example C reactive protein (CRP), IL-6] and preeclampsia. Thirty studies were found on preeclampsia and CRP. In 26 of them, there was a significant relationship between preeclampsia and CRP levels but in four studies no significant relationship was found. They observed a total of 63 studies so far investigating the relationship between IL-6 and preeclampsia. In 55 of them, the results were significantly high, while in eight of them there were no significant differences (11). For this reason, we attribute the marked increase in CRP and IL6 levels to COVID-19 infection. According to them, elevated IL-6 and CRP levels may also be elevated due to COVID-19 infection and preeclampsia. The very high level of IL-6 is thought to be due to the cytokine storm observed at COVID-19 (12).

There are studies that indicate that interleukin-6 can effectively assess disease severity and predict outcomes in patients with COVID-19. While being infected we also found elevated IL-6 levels, after the recovery levels went back to the normal range, so our case also confirms that IL-6 is a good marker in COVID-19 disease (12).

PCT is now generally accepted as a good diagnostic marker of the inflammatory process. The serum PCT levels rise more rapidly than C reactive protein (CRP) levels and peak within a very short time. If the patient responds appropriately to the treatment, the level of PCT returns to the normal range faster than CRP. PCT plasmatic levels are increased in preeclampsia and its levels correlate with the severity of the disease (13). Our case supports the observation of Montagnana et al. that CRP levels are high in severe preeclampsia (proteinuria > 300 mg/day, systolic blood pressure > 140 mmHg, and diastolic blood pressure > 90 mmHg) (13).

Neutrophil-Lymphocyte Ratio (NLR) was significantly associated with the development of death in patients with COVID-19 (14, 15). NLR is an easily measurable, available, cost-effective, and reliable parameter, which on continuous monitoring could be useful for the diagnosis and treatment of COVID-19. Hence, NLR is a useful biomarker to predict the all-cause mortality of COVID-19. Meta-analyzes also shows that the NLR can be a good marker of disease severity and has predictive value for the outcome. The limit cannot be clearly established, but a severe case above 4.5 seems to be expected (16, 17). Elmaradny and colleagues studied the relationship between NLR and preeclampsia. Their results showed that NLR was increasing with preeclampsia. The optimal cut-off level of NLR in preeclampsia was > 4.47 compared to normal pregnancy (18). In our case, the highest NLR ratio was 9.8, suggesting a poor outcome.

The highest measured temperature of the mother was 38.4°C which correlates with the results of Impey et al. who found that 20% of pregnant women with preeclampsia had a measured body temperature above 37.5°C. This percentage among patients who did not show the symptoms of preeclampsia was 6.9 (19).

The strengths of the described case are that laboratory examinations were performed frequently (every day between the 3rd and the 20th and after that every 3 or 4 days), and other parameters that are frequently measured and studied in preeclampsia (body temperature and blood pressure) were also followed during the case. Since there are a limited number of cases of kidney transplant recipient patients who contracted COVID-19 during their pregnancy the uniqueness of the described case makes it worthy of publication.

The authors also do maintain that the results of this study should be considered in light of the limitation of single case studies.

The described case highlights that even though preeclampsia and SARS-CoV-2 infection occurred together, a therapy that was started in the early stage of the disease was able to save both the mother’s and the neonate’s life even though the symptoms of preeclampsia and COVID-19 both became more severe during the hospital stay.

## Conclusion

In our case, we presented a 33-year-old kidney transplant recipient primipara who contracted the SARS-CoV-2 virus in the 26th gestational week. Vaccination at the time of infection has not been recommended during pregnancy. In the fourth month after infection, the patient received the first Pfizer vaccination. She also received a second vaccine (Pfizer) 3°weeks later. Even though her symptoms were not described as severe as in the beginning after she was admitted to the hospital quantitative proteinuria was measured, prior to her admission superimposed hypertension was detected, thus forming the diagnosis of superimposed preeclampsia.

Due to the worsening ultrasound flowmetric results, in the 29th week, prophylactic cesarean delivery was performed.

In conclusion, a therapy that starts in the early phase of COVID-19 disease and preeclampsia and a well-timed prophylactic cesarean section contributed to the positive outcomes.

## Data availability statement

The original contributions presented in the study are included in the article/Supplementary material, further inquiries can be directed to the corresponding author.

## Ethics statement

Written informed consent was obtained from the individual(s) for the publication of any potentially identifiable images or data included in this article.

## Author contributions

ZK, SV, and LW contributed to conception and design of the study. DS collected data on pregnant women infected with COVID-19. DS, BM, DG, MN, and ZK organized the database. SV, BM, LW, and ZK performed the analysis. BM and DS wrote the first draft of the manuscript. All authors contributed to manuscript revision, read, and approved the submitted version.

## References

[1] Shah S Venkatesan RL Gupta A Sanghavi MK Welge J Johansen R Pregnancy outcomes in women with kidney transplant: Metaanalysis and systematic review. *BMC Nephrol.* (2019) 20:24. 10.1186/s12882-019-1213-5 30674290 PMC6345071

[2] Webster P Lightstone L McKay DB Josephson MA. Pregnancy in chronic kidney disease and kidney transplantation. *Kidney Int.* (2017) 91:1047–56. 10.1016/j.kint.2016.10.045 28209334

[3] Rodionov RN Biener A Spieth P Achleitner M Hölig K Aringer M Potential benefit of convalescent plasma transfusions in immunocompromised patients with COVID-19. *Lancet Microbe.* (2021) 2:e138. 10.1016/S2666-5247(21)00030-6 33817676 PMC8009633

[4] Bellos I Pergialiotis V. Risk of pregnancy complications in living kidney donors: A systematic review and meta-analysis. *Eur J Obstet Gynecol Reprod Biol.* (2022) 270:35–41. 10.1016/j.ejogrb.2021.12.037 35016135

[5] Villar J Ariff S Gunier RB Thiruvengadam R Rauch S Kholin A Maternal and neonatal morbidity and mortality among pregnant women with and without COVID-19 infection: The INTERCOVID multinational cohort study. *JAMA Pediatr.* (2021) 175:817–26. 10.1001/jamapediatrics.2021.1050 33885740 PMC8063132

[6] Papageorghiou AT Deruelle P Gunier RB Rauch S García-May PK Mhatre M Preeclampsia and COVID-19: Results from the INTERCOVID prospective longitudinal study. *Am J Obstet Gynecol.* (2021) 225:.e1–289. 10.1016/j.ajog.2021.05.014 34187688 PMC8233533

[7] Huntley BJF Huntley ES Di Mascio D Chen T Berghella V Chauhan SP. Rates of maternal and perinatal mortality and vertical transmission in pregnancies complicated by severe acute respiratory syndrome coronavirus 2 (SARS-Co-V-2) infection: A systematic review. *Obstet Gynecol.* (2020) 136:303–12. 10.1097/AOG.0000000000004010 32516273

[8] Ionescu M Stoian AP Rizzo M Serban D Nuzzo D Mazilu L The role of endothelium in COVID-19. *Int J Mol Sci.* (2021) 22:11920. 10.3390/ijms222111920 34769350 PMC8584762

[9] Bernard I Limonta D Mahal LK Hobman TC. Endothelium infection and dysregulation by SARS-CoV-2: Evidence and caveats in COVID-19. *Viruses.* (2020) 13:29. 10.3390/v13010029 33375371 PMC7823949

[10] Nardo AD Schneeweiss-Gleixner M Bakail M Dixon ED Lax SF Trauner M. Pathophysiological mechanisms of liver injury in COVID-19. *Liver Int.* (2021) 41:20–32. 10.1111/liv.14730 33190346 PMC7753756

[11] Guney G Taskin MI Tokmak A. Increase of circulating inflammatory molecules in preeclampsia, an update. *Eur Cytokine Netw.* (2020) 31:18–31. 10.1684/ecn.2020.0443 32540805

[12] Liu F Li L Xu M Wu J Luo D Zhu Y Prognostic value of interleukin-6, C-reactive protein, and procalcitonin in patients with COVID-19. *J Clin Virol.* (2020) 127:104370. 10.1016/j.jcv.2020.104370 32344321 PMC7194648

[13] Montagnana M Lippi G Albiero A Scevarolli S Salvagno GL Franchi M Procalcitonin values in preeclamptic women are related to severity of disease. *Clin Chem Lab Med.* (2008) 46:1050–1. 10.1515/CCLM.2008.199 18624624

[14] Jimeno S Ventura PS Castellano JM García-Adasme SI Miranda M Touza P Prognostic implications of neutrophil-lymphocyte ratio in COVID-19. *Eur J Clin Invest.* (2021) 51:e13404. 10.1111/eci.13404 32918295

[15] Wang X Li X Shang Y Wang J Zhang X Su D. Ratios of neutrophil-to-lymphocyte and platelet-to-lymphocyte predict all-cause mortality in inpatients with coronavirus disease 2019 (COVID-19): A retrospective cohort study in a single medical centre. *Epidemiol Infect.* (2020) 148:e211. 10.1017/S0950268820002071 32900409 PMC7506174

[16] Sarkar S Khanna P Singh AK. The impact of neutrophil-lymphocyte count ratio in COVID-19: A systematic review and meta-analysis. *J Intensive Care Med.* (2022) 37:857–69. 10.1177/08850666211045626 34672824 PMC9160638

[17] Zhang S Guo M Duan L Wu F Hu G Wang Z Development and validation of a risk factor-based system to predict short-term survival in adult hospitalized patients with COVID-19: A multicenter, retrospective, cohort study. *Crit Care.* (2020) 24:438. 10.1186/s13054-020-03123-x 32678040 PMC7364297

[18] Elmaradny E Alneel G Alkhattaf N AlGadri T Albriakan N. Predictive values of combined platelet count, neutrophil-lymphocyte ratio, and platelet-lymphocyte ratio in preeclampsia. *J Obstet Gynaecol.* (2021) 42:1011–17 10.1080/01443615.2021.1986476 34927550

[19] Impey L Greenwood C Sheil O MacQuillan K Reynolds M Redman C. The relation between pre-eclampsia at term and neonatal encephalopathy. *Arch Dis Child Fetal Neonatal Ed.* (2001) 85:F170–2. 10.1136/fn.85.3.f170 11668157 PMC1721320

