# Supplementary material for: Case report: COVID-19 infection in a pregnant 33-year-old kidney transplant recipient
Source: Front Med (Lausanne). 2022 Aug 30;9:948025. doi: 10.3389/fmed.2022.948025 (PMC9468219; doi:10.3389/fmed.2022.948025)
Supplement: Supplementary file 1 [file Data_Sheet_1.docx]

|  | **Relevant laboratory findings before, during, and after the pregnancy and the COVID-19 infection** | | | | | | | | | | | | |
| --- | --- | --- | --- | --- | --- | --- | --- | --- | --- | --- | --- | --- | --- |
|  | **normal ranges** | **35 days prior to hospital admission** | **4 days prior to hospital admission (positive COVID-19 test result)** | **Day 1 (hospital admission)** | **Day 7** | **Day 11** | **Day 15 (birth)** | **Day 20** | **Day 24** | **Day 42** | **Day 63 (discharged from hospital)** | **88 days after being discharged from the hospital** | **236 days after being discharged from the hospital** |
| **WBC** | 4.5-11 (G/L) | 13.04 | 9.48 | 13.63 | 12.05 | 9.16 | 12.8 | 14.14 | 10.79 | 11.75 | 8.86 | 5.5 | 6.67 |
| **% of neutrophyles** | 45.0-70.0 (%) | 70.1 | 72.9 | 60 | 88.2 | 66.3 | 69.3 | 68 | 56.2 | 61.1 | 51 | 50.4 | 67.1 |
| **% of leukocytes** | 25.0-45.0 (%) | 24.2 | 19.5 | 33.4 | 9 | 28.8 | 25.6 | 26.4 | 36.2 | 33 | 41 | 39.1 | 25.5 |
| **Platelet count** | 150-400 (G/L) | 220 | 153 | 148 | 104 | 129 | 167 | 220 | 379 | 241 | 269 | 263 | 249 |
| **LDH** | <248 (U/L) | - | - | - | 309 | 266 | 287 | 271 | 229 | 189 | 199 | - | - |
| **eGFR** | 90-120 (mL/min/1.73 m^2^) | 54.3 | 36.8 | 39.9 | 33 | 37.4 | 48.1 | 49.8 | 59.2 | 59.2 | 46.3 | 41.3 | 55.4 |
| **Uric Acid** | 154-357 (µmol/L) | 460 | 582 | - | 640 | - | 523 | 496 | - | - | 484 | 438 | 394 |
| **BUN** | 2.8-7.2 (mmol/L) | 6.9 | 11 | 9.1 | 9.5 | 10.4 | 8.3 | 4.9 | 6.2 | 8.2 | 7.9 | 8.4 | 6.2 |
| **Creatinin** | 45-84 (mmol/L) | 114 | 157 | 147 | 172 | 155 | 126 | 122 | 106 | 128 | 130 | 143 | 112 |
| **Total protein, Blood** | 66-83 (g/L) | 63.4 | 62 | 62.3 | 53.9 | - | 54.6 | 61.4 | 59.2 | 64 | 63.7 | - | 74.1 |
| **Albumin, Blood** | 35-52 (g/L) | 35.6 | 33.5 | - | 27 | - | 24.9 | 29.3 | 29.3 | 35.8 | 37 | - | 41.8 |
| **Total protein, Urine** | <80 (mg/L) | - | - | - | - | 1317 | 1075 | 1259 | - | 713 | 672 | 280 | 202.6 |
| **ASAT** | <35 (U/L) | 18 | - | 25 | 30 | - | 135 | 61 | - | 21 | 26 | - | 18 |
| **ALAT** | <35 (U/L) | 14 | - | 17 | 33 | - | 167 | 122 | - | 36 | 42 | - | 11 |
| **GGT** | <38 (U/L) | 8 | - | 16 | 111 | - | 109 | 80 | - | 29 | 21 | - | 10 |
| **CRP** | <5.00 (mg/L) | - | - | 3.6 | 69.9 | 47.5 | 2.9 | 30.5 | 1.7 | 0.3 | 0.3 | - | - |
| **PCT** | <0.50 (µg/L) | - | - | 0.14 | 0.53 | 0.6 | 0.47 | 0.21 | 0.05 | 0.04 | - | - | - |
| **Ferritin** | 10-120 (µg/L) | - | - | - | 241 | 408 | 365 | 260 | - | - | - | - | - |
| **IL-6** | <7 (pg/mL) | - | - | - | 23.6 | 7.4 | 4.12 | 17.5 | 6.76 | 3.84 | - | - | - |

| **Ultrasound results during hospital stay** | | | | | | | | |
| --- | --- | --- | --- | --- | --- | --- | --- | --- |
|  | **Day 5** | **Day 7** | **Day 8** | **Day 9** | **Day 12** | **Day 13** | **Day 14** | **Day 15 (birth)** |
| **AFI (cm)** |  |  |  |  |  | 13 |  | 14 |
| **BPD (mm)** |  | 64 |  |  |  | 65 |  | 65 |
| **HC (mm)** |  | 247 |  |  |  | 245 |  | 240 |
| **AC (mm)** |  | 243 |  |  |  | 221 |  | 230 |
| **FL (mm)** |  | 48 |  |  |  | 47 |  | 49 |
| **Humerus (mm)** |  |  |  |  |  |  |  | 44 |
| **FHR** | 144 | 134 | 158 | 141 |  | 138 | 154 | 145 |
| **Estimated Weight** |  | 930 |  |  |  | 821 |  | 1004 |
| **Umbilical Artery PI** | 0.91 | 1.19 | 1.13 | 0.69 | 1.98 | 0.78 | 1.59 | total diastolic stop |
| **Umbilical Artery RI** | 0.56 | 0.69 | 0.68 | 0.47 | 1 | 0.68 | 0.79 | total diastolic stop |
| **Middle Cerebral Artery (MCA) PI** | 1.34 | 1.89 | 1.53 | 1.17 | 1.96 | 0.96 | 0.96 | 1.58 |
| **Middle Cerebral Artery (MCA) RI** | 0.73 | 0.9 | 0.78 | 0.74 | 1 | 0.73 | 0.79 | 0.86 |
| **CPR** | 1.47 | 1.588 |  |  | -3.272 |  |  |  |
| **Comments** |  |  |  |  | *starting of circulation centralization* |  | *circulation centralization* | *pathological flow* |

|  | **Highest daily blood pressure** | | | | | | | | | | | |
| --- | --- | --- | --- | --- | --- | --- | --- | --- | --- | --- | --- | --- |
|  | **Day 1 (hospital admission)** | **Day 2** | **Day 5** | **Day 9** | **Day 12** | **Day 13** | **Day 14** | **Day 15 (birth)** | **Day 16** | **Day 17** | **Day 22** | **Day 29** |
| **systolic blood pressure (mmHg)** | 169 | 176 | 156 | 131 | 140 | 130 | 141 | 158 | 158 | 147 | 130 | 125 |
| **dyastolic blood pressure (mmHg)** | 93 | 119 | 114 | 95 | 105 | 85 | 103 | 117 | 120 | 100 | 85 | 85 |
|  | **Average daily blood pressure** | | | | | | | | | | | |
| **systolic blood pressure (mmHg)** | 169 | 158 | 141 | 124 | 136 | 129 | 134 | 147 | 151 | 134 | 130 | 125 |
| **dyastolic blood pressure (mmHg)** | 93 | 100 | 102 | 88 | 99 | 88 | 95 | 109 | 106 | 94 | 85 | 85 |

On the supplementary tables relevant laboratory findings before, during, and after the pregnancy and the COVID-19 infection, ultrasound results during hospital stay, highest daily blood pressures and average daily blood pressures are visible.
