# Supplementary figures and images for: Case report: COVID-19 infection in a pregnant 33-year-old kidney transplant recipient
Source: Front Med (Lausanne). 2022 Aug 30;9:948025. doi: 10.3389/fmed.2022.948025 (PMC9468219; doi:10.3389/fmed.2022.948025)

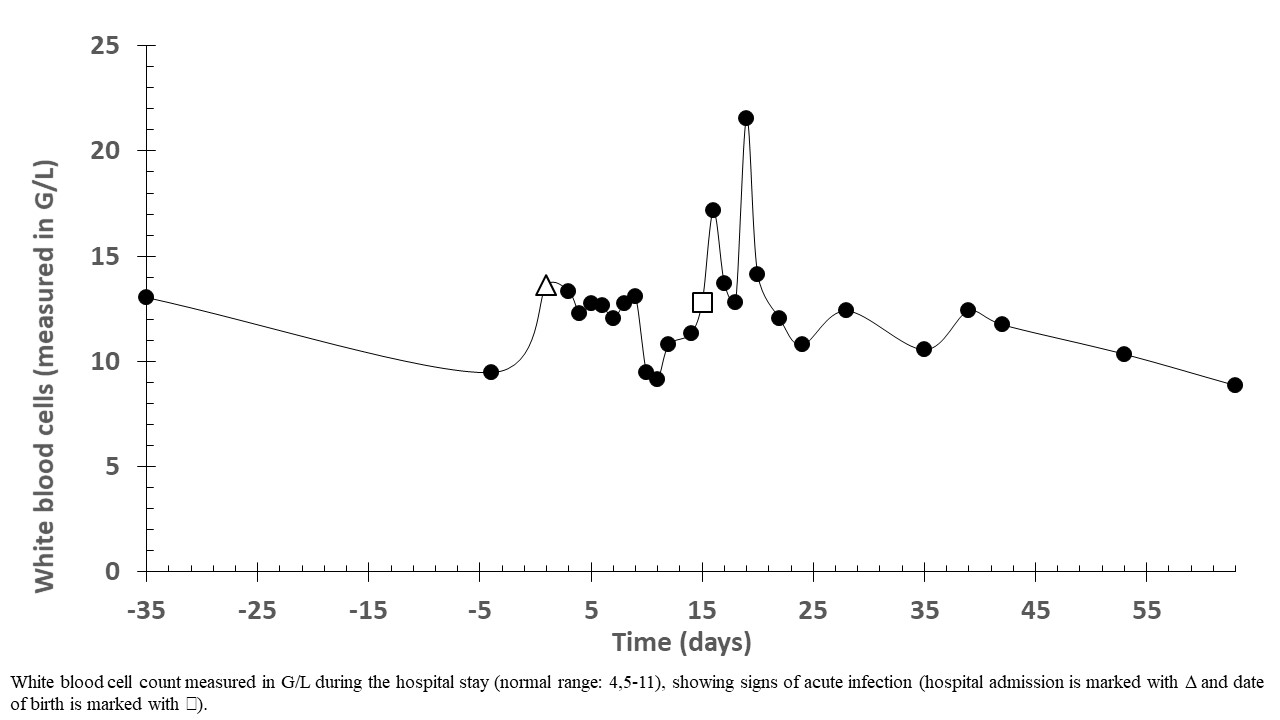

Supplement: Supplementary file 2 [file Image_1.JPEG]

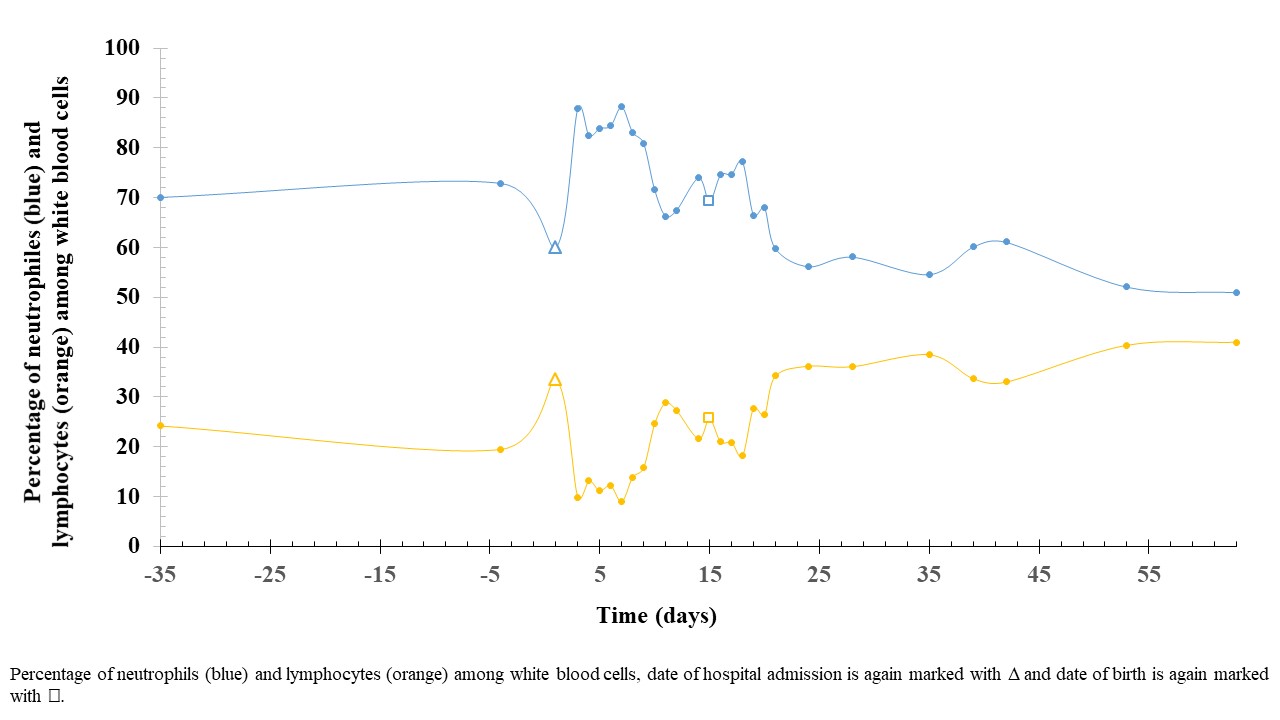

Supplement: Supplementary file 3 [file Image_2.JPEG]

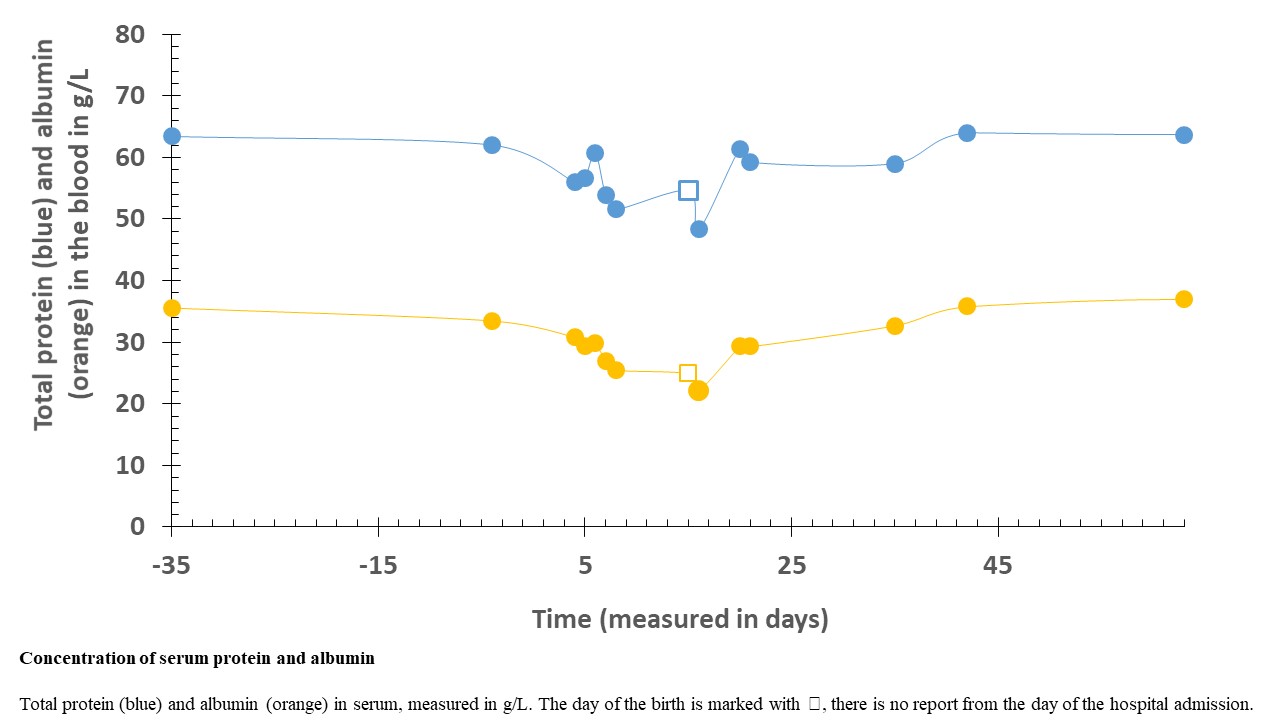

Supplement: Supplementary file 4 [file Image_3.JPEG]
